# Supplementary material for: Framing the Family: A Qualitative Exploration of Factors That Shape Family-Level Experience of Pediatric Genomic Sequencing
Source: Children (Basel). 2023 Apr 25;10(5):774. doi: 10.3390/children10050774 (PMC10217651; doi:10.3390/children10050774)
Supplement: Supplementary file 1 [file children-10-00774-s001.zip › children-2264830-supplementary.pdf]

Framing the family: A qualitative exploration of factors that shape family-level experience of pediatric genomic sequencing

Supplementary Material

Authors: Hadley Stevens Smith, PhD, MPSA,[1] Emily S. Bonkowski, ScM,[2,3] Madison R. Hickingbotham, MS,[1] Raymond Belanger Deloge, MS,[4] Stacey Pereira, PhD[5]

[1]PRecisiOn Medicine Translational Research (PROMoTeR) Center, Department of Population Medicine, Harvard Medical School and Harvard Pilgrim Health Care Institute, Boston, MA 02215, USA
[2]Institute for Public Health Genetics, University of Washington School of Public Health, Seattle, WA 98195, USA
[3]Center for Pediatric Neurological Disease Research, St. Jude Children's Research Hospital, Memphis, TN 38105, USA
[4]Department of Molecular and Human Genetics, Baylor College of Medicine, Houston, TX 77030, USA
[5]Center for Medical Ethics and Health Policy, Baylor College of Medicine, Houston, TX 77030, USA

Table S1. Patients' clinical diagnoses

**Patient's primary clinical diagnosis, n (%)**	
**Social and Behavioral**	
Autism Spectrum Disorder	5 (12.2%)
Attention and Concentration Deficit	1 (2.4%)
**Developmental Delay**	
Cognitive and Neurobehavioral Dysfunction	1 (2.4%)
Delayed Milestones	1 (2.4%)
Developmental Delay	2 (4.9%)
Global Developmental Delay	4 (9.8%)
Gross Motor Development Delay	1 (2.4%)
Intellectual Delay	1 (2.4%)
Intellectual Disability	2 (4.9%)
Speech Delay	2 (4.9%)
**Dysmorphic Craniofacial Features**	
Dysmorphic Features	1 (2.4%)
Plagiocephaly	1 (2.4%)
**Spasm/Seizure Disorder**	
Seizure Disorder	1 (2.4%)
**Musculoskeletal/Connective Tissue**	
Ehlers-Danlos syndrome	1 (2.4%)
Spastic Diplegia	1 (2.4%)
Toe Walking	1 (2.4%)
**Multisystemic Disorders**	
47, XYY	1 (2.4%)
Cornelia de Lange syndrome	1 (2.4%)
Hypohidrotic Ectodermal Dysplasia	1 (2.4%)
MN1 C-Terminal Truncation syndrome (CEBALID syndrome)	1 (2.4%)
Noonan syndrome-like disorder	1 (2.4%)
Williams Syndrome	1 (2.4%)
**Gastrointestinal/Genitourinary**	
Posterior Urethral Valves	1 (2.4%)
Infantile Liver Failure Syndrome Type 1	1 (2.4%)

**Cardiac**	
Tetralogy of Fallot	1 (2.4%)
Atrial Septal Defect	1 (2.4%)
**Other**	
Oral Aversion	1 (2.4%)
Overgrowth syndrome	1 (2.4%)
Pyruvate Dehydrogenase Deficiency	1 (2.4%)
Retinitis Pigmentosa of Both Eyes	1 (2.4%)
Sensorineural Hearing Loss of Both Ears	1 (2.4%)
**Patient's secondary clinical diagnosis**	
**Social and Behavioral**	
Autism Spectrum Disorder	1 (2.4%)
Attention Deficit Hyperactivity Disorder	2 (4.9%)
**Developmental Delay**	
Cognitive communication deficit	1 (2.4%)
Developmental delay	3 (7.3%)
Expressive language delay	1 (2.4%)
Mixed receptive expressive language disorder	1 (2.4%)
Mixed receptive language disorder	1 (2.4%)
Speech and language deficits	1 (2.4%)
Speech articulation disorder	1 (2.4%)
Speech delay	1 (2.4%)
Family history of speech and language disorder	1 (2.4%)
Global developmental delay	1 (2.4%)
**Dysmorphic Craniofacial Features**	
Dysmorphic craniofacial features	2 (4.9%)
Macrocephaly	2 (4.9%)
**Spasm / Seizure Disorder**	
Infantile spasms	2 (4.9%)
**Musculoskeletal / Connective Tissue**	
Hypermobility syndrome	1 (2.4%)
Hypotonia	2 (4.9%)
Hypertonia	1 (2.4%)
Short stature	1 (2.4%)
Action tremor	1 (2.4%)
Right sided hemiplegic cerebral palsy	1 (2.4%)
**Gastrointestinal/Genitourinary**	
Constipation, unspecified constipation type	1 (2.4%)
Penile hypospadias	1 (2.4%)
Congenital choanal atresia	1 (2.4%)
**Other**	
Family history of SIDS	1 (2.4%)
Juvenile myelomonocytic leukemia	1 (2.4%)
Polymicrogyria	1 (2.4%)
Bronchopulmonary dysplasia	1 (2.4%)
Dyslexia	1 (2.4%)
